# Assessment of Maternal Effects and Genetic Variability in Resistance to *Verticillium dahliae* in Olive Progenies

**DOI:** 10.3390/plants10081534

**Published:** 2021-07-27

**Authors:** Pedro Valverde Caballero, Carlos Trapero Ramírez, Diego Barranco Navero, Francisco J. López-Escudero, Ana Gordon Bermúdez-Coronel, Concepción Muñoz Díez

**Affiliations:** Excellence Unit ‘María de Maeztu’ 2020-23, Department of Agronomy, ETSIAM, University of Córdoba, 14071 Córdoba, Spain; g82vacap@uco.es (P.V.C.); carlostrapero@uco.es (C.T.R.); dbarranco@uco.es (D.B.N.); ag2loesj@uco.es (F.J.L.-E.); anagordonbc@gmail.com (A.G.B.-C.)

**Keywords:** disease, olive breeding, reciprocal crosses, resistance, *Verticillium dahliae*

## Abstract

The use of genetic resistance is likely the most efficient, economically convenient and environmentally friendly control method for plant diseases, as well as a fundamental piece in an integrated management strategy. This is particularly important for woody crops affected by diseases in which mainly horizontal resistance mechanisms are operative, such as Verticillium wilt, caused by *Verticillium dahliae*. In this study, we analyzed the variability in resistance to Verticillium wilt of olive trees in progenies from five crosses: ‘Picual’ × ‘Frantoio’, ‘Arbosana’ × ‘Koroneiki’, ‘Sikitita’ × ‘Arbosana’, ‘Arbosana’ × ‘Frantoio’ and ‘Arbosana’ × ‘Arbequina’ and their respective reciprocal crosses. Additionally, seedlings of ‘Picual’ and ‘Frantoio’ in open pollination were used as controls. In October 2016 and 2018, the fruits were harvested, and seeds germinated. Six-week-old seedlings were inoculated by dipping their bare roots in a conidial suspension of *V. dahliae,* and disease progress in terms of symptom severity and mortality was evaluated weekly. Additionally, seedling growth was evaluated every two weeks. At the end of the experiment, no significant differences were found for any of the assessed parameters when reciprocal crosses were compared. These results suggest that there is no maternal or paternal effect in regard to the heritability of resistance. In addition, this study identifies the best crosses for obtaining the highest number of resistant genotypes, highlighting the importance of the selection of specific cultivars to optimize the breeding process.

## 1. Introduction

Verticillium wilt of olive trees (VWO), caused by the soil pathogen *Verticillium dahliae*, Kleb., is currently considered the most destructive disease in olive orchards in Spain, the largest olive oil producer country, as well as in most olive-growing regions worldwide [1,2]. The impact of this disease has increased in recent decades due to the establishment of new olive plantations in fertile soils previously cultivated with host crops of the pathogen, mainly cotton and vegetables. To effectively control the disease, an integrated management strategy is needed, since none of the available measures is effective when applied individually. This strategy includes preventive measures applied before planting, such as the use of pathogen-free plants and soils, and measures after planting, principally aimed at preventing the introduction of the pathogen or reducing its increase and efficacy [3]. In this context, the use of genetically resistant genotypes is probably the most important measure, and many studies have attempted to identify sources of resistance [4,5]. Most olive cultivars evaluated to date are susceptible to the disease, whereas a few cultivars, such as ‘Frantoio’, ‘Changlot Real’ and ‘Empeltre’, have shown high levels of resistance [6,7,8].

Using the most resistant cultivars mentioned above as genitors, several studies have focused on evaluating the level of this character in progenies coming from different crosses. Root-dipping inoculation of young seedlings has proven to be the most reliable method for the identification of resistance [9]. Interestingly, there has been wide variability in the level of resistance in the progenies, even finding resistant genotypes in progenies from crosses in which both genitors were susceptible [10,11]. However, the resistance level of the genitors defined the percentage of resistant genotypes within each progeny; therefore, the most resistant parents generated the highest percentage of resistant seedlings. Trapero et al. (2015) [11] evaluated the resistance to VWO in a large progeny of ‘Frantoio’ × ‘Picual’ and its reciprocal crossing (‘Picual’ × ‘Frantoio’) and discussed that there could be differences in the percentage of resistant individuals depending on the direction of the cross, pointing out the involvement of some form of asymmetrical inheritance.

Different genetic phenomena can participate in the asymmetrical inheritance of an agronomic trait: maternal effect and cytoplasmic inheritance. The maternal effect has been defined as the causal influence of the maternal genotype or phenotype on the offspring phenotype, while cytoplasmic inheritance is organelle inheritance via the egg [12]. Following this rule of thumb, the maternal effect has been related to different traits in several plant species, such as drought tolerance or root weight in sweet potato [13,14], seedling vigor in maize [15] or tuber yield in potato [16].

On the other hand, three classes of maternal effects have been identified: cytoplasmic genetic, endosperm nuclear and maternal phenotypic effects. Several studies have shown that variation in seed, seedling and adult traits caused by maternal effects can have important consequences on the seedling response to different treatments [17,18].

There are several mechanisms involved in the asymmetrical heritability of different agronomic traits in sexual reproduction. For example, imprinting (a type of parent-of-origin effect) is an epigenetic phenomenon where one allele is expressed over the other depending on the sex of the parent that contributed the allele [19]. Imprinting is common in flowering plants and has been mostly related to endosperm tissue, although other authors have found one imprinted gene (maternally expressed in the embryo 1 gene) in both embryo and endosperm [20].

Furthermore, only maternal transcript sequences were detected in both progenies resulting from reciprocal crosses, which were correlated with differential allelic methylation [21]. Examples of imprinting are the irregular distribution of anthocyanin in the aleurone layer of maize endosperm [22] and the control of the germination process in *Arabidopsis* seeds [23]. However, no relationship has been established between imprinting and disease resistance in plants thus far.

To the best of our knowledge, no information regarding maternal or paternal effects has been published specifically in olive crops, and only a few studies in other plant species have been conducted. Among the latter, we can find cases of resistance to southern corn blight (*Cochliobolus heterostrophus*) and yellow corn blight (*Mycosphaerella zeae-maydis*) in maize (*Zea mays*), both associated with maternally inherited T male-sterile cytoplasm [24]. Additionally, the inheritance of resistance to anthracnose, a disease caused by the fungal pathogen *Colletotrichum dematium*, was determined largely by a nonnuclear, additive paternal effect in *Ipomoea purpurea* [25]. Furthermore, a study to develop rice cultivars resistant to bacterial blight (caused by *Xanthomonas oryzae* pv. oryzae) found that maternal contribution was important in controlling the virulence of this disease [26]. Interestingly, Vivas et al. [27] demonstrated that abiotic differences in the maternal environment affected both plant growth and resistance to *Fusarium circinatum* traits in the subsequent generation in *Pinus pinaster*. Conversely, maternal effects or cytoplasmic inheritance were less influential when ten bean parental lines (*Vicia faba* L.) were evaluated for their resistance to chocolate spot disease caused by *Botrytis fabae* [28]. Similar results were obtained when maternal and cytoplasmic effects were evaluated on northern corn leaf blight (caused by the heterothallic ascomycete *Setosphaeria turcica*), the most devastating leaf pathogen in maize [29].

Although studies of genetic resistance or susceptibility are crucial in devising a viable strategy for current breeding programs in plants, the evaluation of possible asymmetrical heritability on diseases has been scarce and inconsistent. The lack of full-diallel mating designs, which include reciprocal crosses, has limited the information regarding this particular field. In this context, the main goal of this study was to generate and evaluate large olive progenies from reciprocal crosses to (a) assess the existence of possible maternal or paternal effects on seed germination capacity and resistance to *Verticillium dahliae* and (b) explore the best genitor crosses with regard to offspring resistance and good germination to optimize the olive-breeding program process.

## 2. Results

The genotypes from the crosses ‘Arbosana’ × ‘Picual’, ‘Sikitita’ × ‘Frantoio’ and their respective reciprocal pairs were eliminated from the experiments, as they were classified as incompatible crosses in paternity testing.

### 2.1. Seed Germination Rate

One thousand nine hundred ninety-two seeds derived from twelve different crosses germinated in two years, 2016 and 2018. The average germination in the two years was 52.2%. The germination rate in 2016 significantly varied between crosses, being 29.8% for the crossing of ‘Arbosana’ × ‘Sikitita’ and ‘Arbosana’ × ‘Arbequina’ and 80.6% for ‘Picual’ in free pollination (Table 1). In 2018, germination ranged between 23.7% in ‘Sikitita’ × ‘Arbosana’ and 80.6% in ‘Picual’ under free pollination. There were no significant differences in germination rate between any of the reciprocal crosses in either year (Table 1).

### 2.2. Symptom and Disease Parameters

**Figure 1:**
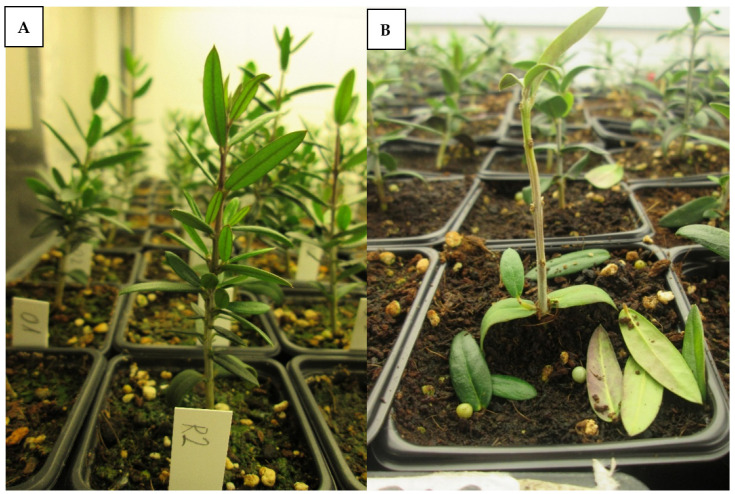
Olive genotypes growing in the climatic chamber: (**A**) Control plant without symptoms; and (**B**) typical observed symptoms (green defoliation) in an inoculated genotype during the evaluation.

Approximately three months after germination, the seedlings were successfully infested by the pathogen by root dipping in a conidial suspension (Figure 1).

Symptom onset was first observed the fourth week after inoculation and consisted of green defoliation, purple discoloration in leaves, yellowing, total or partial necrosis and lack of growth. We confirmed that these symptoms were caused by *Verticillium dahliae* by performing isolations in Petri dishes with PDA (potato dextrose agar) and verifying fungal growth in all isolations.

**Figure 2:**
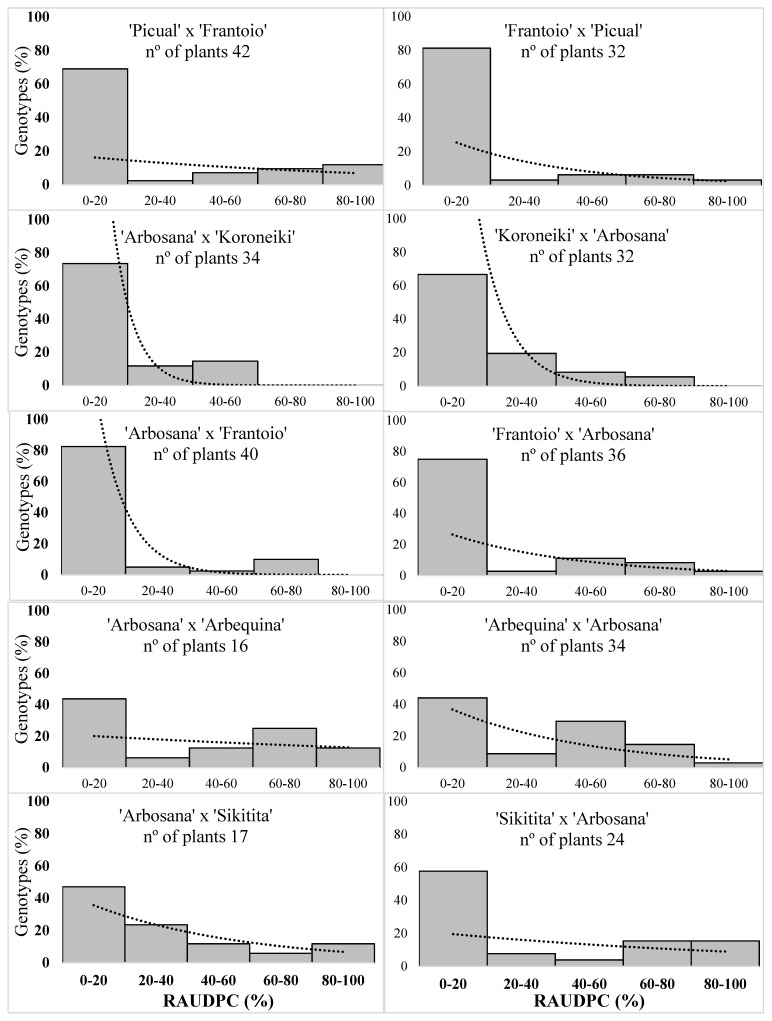
Frequency histogram of the relative area under the disease progress curve (RAUDPC) with their respective exponential tendency line of the reciprocal crosses tested in 2016.

**Figure 3:**
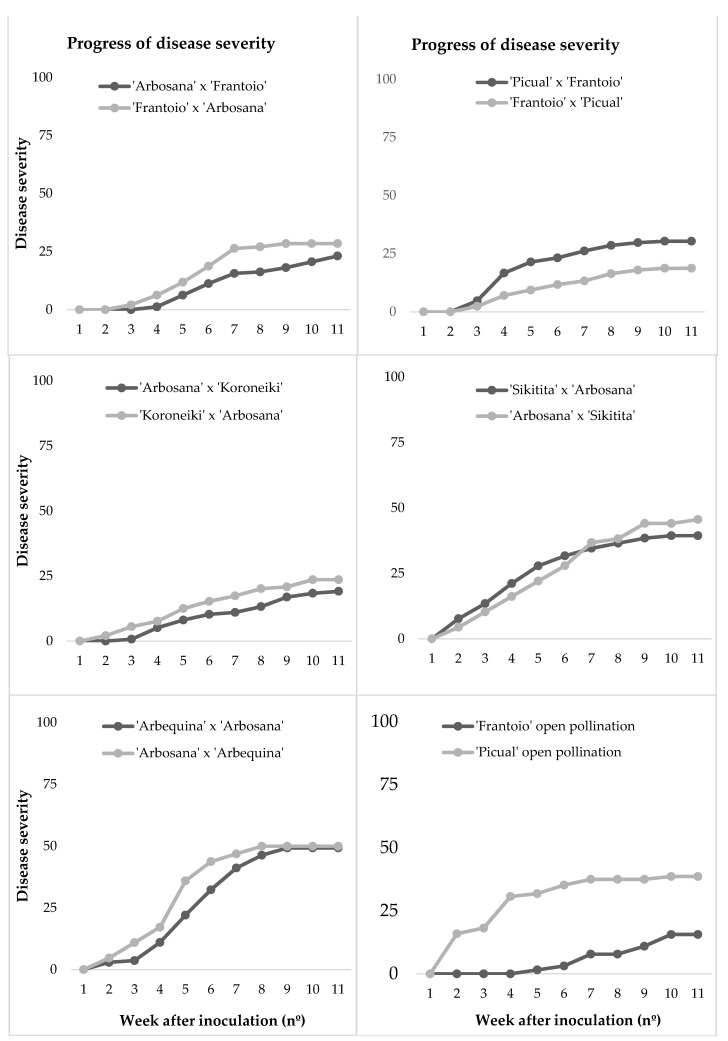
Disease severity over time in the reciprocal crosses conducted in 2016 and inoculated with *V. dahliae*.

In 2016, the disease incidence (DI) of the seedlings ranged between 25% in the offspring of ‘Frantoio’ in open pollination and 62.5% in the offspring of ‘Arbosana’ × ‘Arbequina’, with the average DI in all crossings being 43.1% (Table 2). The value of the RAUDPC (relative area under the disease progress curve) varied between 10.4% in the offspring of ‘Arbosana’ × ‘Koroneiki’ and 38.1% in the offspring of ‘Arbosana’ × ‘Arbequina’ (Figure 2). Mortality (M) values also ranged between 2.9% in ‘Arbosana’ × ‘Koroneiki’ and 43.8% in the offspring of ‘Arbosana’ × ‘Arbequina’, with an average of 21.4% (Table 2). Disease parameters revealed high variability among progenies. However, no significant differences were found when we performed pairwise comparisons between reciprocal crosses according to their phytopathological variables. This fact was highlighted by the progress of disease severity; in Figure 3, it can be observed how the curves belonging to the reciprocal crosses have the same slope and conclude almost at the same point. We only found clear significant differences when comparing the crosses ‘Picual’ and ‘Frantoio’ in open pollination, also with significant differences in RAUDPC and final severity (Table 2).

The evaluation in 2018 was performed to confirm the patterns previously observed in reciprocal crossings involving different cultivars. The highest values in all evaluated parameters were obtained by ‘Arbosana’ × ‘Sikitita’, with the exception of mortality (M), in which the highest value was obtained by ‘Sikitita’ × ‘Arbosana’. In contrast, ‘Frantoio’ in open pollination showed the lowest values for all evaluated parameters. Corroborating the results obtained in 2016, no significant differences in disease parameters were obtained when we performed a pairwise comparison between each reciprocal progeny.

### 2.3. Seedling Growth

We found significant differences in growth increase (GI) when inoculated seedlings with no visible symptoms and noninoculated (control) seedlings were compared (Table 1). The average GI in nonaffected inoculated genotypes (no symptoms) ranged from 9.8 cm in the offspring of ‘Koroneiki’ × ‘Arbosana’ to 16.5 cm in ‘Sikitita’ × ‘Arbosana’, with clear significant differences between them. The increase in average growth in control plants did not show differences between reciprocal crosses.

### 2.4. Germination Rate and Resistance Level among Progenies

Since we found no significant differences between any of the reciprocal crosses attending to their phytopathological values, we merged both in a single unit (Table 3). Afterwards, we compared the germination rate and resistance level among the different crosses, taking advantage of the greater sample size of each cross.

The results confirmed the high variability among olive crossings in all study variables. Regarding germination, the genotypes coming from the crosses with ‘Arbosana’ and ‘Arbequina’ showed the lowest value (36.1%). On the other hand, the crosses with ‘Picual’ and ‘Frantoio’ had the best germination values (69.2%) (Table 3).

Regarding the resistance to Verticillium wilt, we observed two groups: first, the crosses involving the resistant cultivars ‘Koroneiki’ and ‘Frantoio’, which gave rise to the highest percentage of resistant offspring in terms of DI, M and RAUDPC; and second, the crosses that only had ‘Arbosana’, ‘Sikitita’ and ‘Picual’ as genitors, which generated a larger number of susceptible offspring. For example, the cross with the lowest mortality value (4.3%) was ‘Arbosana’ and ‘Koroneiki’, whereas those reciprocal crosses of ‘Arbosana’ and ‘Arbequina’ showed the highest values of DI (62%), final disease severity (50%), M (38%) and RAUDPC (33.1%).

## 3. Discussion

In recent decades, olive cultivation has undergone dramatic changes, mainly due to the intensification of plantation systems and the incorporation of irrigation. In many cases, new olive orchards have occupied fertile lands in river valleys previously cultivated with other species [30]. Some of these species, particularly cotton and vegetables, are hosts of *V. dahliae;* therefore, soils are heavily infested with this pathogen [8]. This situation has given rise to an unprecedented incidence of Verticillium Wilt of Olive trees (VWO) that has been aggravated since no resistant cultivars are available for its control. Thus, since 2008, studies have focused on finding new olive cultivars resistant to VWO and have adapted to intensive plantation systems [31].

The first step in a breeding program is the selection of genitors that could confer valuable traits to their offspring. In olive trees, out of more than 250 evaluated cultivars [7,8], only the cultivars ‘Frantoio’, ‘Empeltre’ and ‘Changlot Real’ showed wide and solid resistance to VWO, but none of them presented low vigor and an adequate architecture adapted to mechanical harvesting [1]. In addition, how and in what proportion these resistant cultivars are able to transfer resistance to their offspring is not well known. Indeed, it is worth mentioning the wide variability of the resistance level in offspring, even finding resistant genotypes in olive progenies from crosses in which both genitors were susceptible [10,11,32].

The maternal effect has been studied in several crops and for diverse agronomic characteristics. It can constitute a valuable tool in a breeding program to select the most favorable parents so that the character to be improved is present in the greatest possible amount in the offspring [18,28]. One of the most direct quantitative methods to determine if there is a maternal effect in the inheritance of a given trait is using reciprocal crosses. This effect can be dependent on the evaluated trait. For example, Liu et al. [33] demonstrated in *Pyrus* that the inheritance success of some characteristics depends on the cultivar used as a male or female genitor.

The identification of a possible maternal effect on the inheritance of resistance to VWO was the main goal of this study along with the identification of the most effective crosses generating seedlings resistant to this disease. There is little available information on the maternal effect on agronomic characteristics associated with olive trees, and particularly, information on olive diseases is very scarce. Trapero et al. [11] presented data where a tendency toward a higher proportion of resistant plants in the progeny from the cross ‘Frantoio’ × ‘Picual’ than that from ‘Picual’ × ‘Frantoio’ was observed. This study could suggest that when using a resistant cultivar such as a mother, the progeny will be more resistant than in the contrary case. However, in the present study, all phytopathological parameters evaluated indicate that both genitors contribute equally to their offspring resistance to *Verticillium* wilt. The mentioned ‘Frantoio’ × ‘Picual’ and ‘Picual’ × ‘Frantoio’ reciprocal crosses, as well as others, were inoculated and analyzed. These results ease the breeding process since the availability of pollen from a certain cultivar to perform directed crosses is not always guaranteed. This low availability of pollen to use in crosses can be due to the prevalence of some cultivars, among others, low pollen production [34] or variability in flowering time [35].

In this study, we assessed a large number of progenies to determine the germination rate of seedlings and their response to infections caused by *V. dahliae* after inoculation under artificial conditions. For these two traits, we found high variability in the individuals of the progenies of the different crosses, but we did not find significant differences when we compared the results in the pairs of each reciprocal cross. In *Arabidopsis*, in contrast, reciprocal crosses have shown that imprinting plays a role in regulating germination processes and that preferential maternal allelic expression can implement maternal inheritance of seed dormancy levels [23]. In addition, maternal small interfering RNAs that induce RNA-directed DNA methylation are also involved in *Arabidopsis* seed development [36].

Once we discarded the existence of the maternal effect, this study focused on selecting the crosses that maximize the percentage of seedlings resistant to *Verticillium* wilt. Interestingly, one of the demonstrated results from our study was the wide variability in the resistance level of the offspring, which is consistent with previous studies [10,11,32]. In an olive-breeding program, two strategies can be followed. The first is to evaluate in controlled conditions the resistance of a large number of seedlings by artificial inoculations in the first breeding program step, when they have 6 weeks, and then evaluate the agronomic characteristics under field conditions. Following this strategy, resistant genotypes are always found. The second alternative strategy is to select genotypes with good agronomic characteristics under field conditions, perform clonal propagation, and then evaluate their resistance by artificial inoculations and evaluations in infested fields [10,32]. In this last case, as already mentioned, it must be taken into account that the possibility of finding disease-resistant genotypes within these available genotypes of agronomic interest could be low.

In addition, it has been found that some genitors are not very suitable to be included in a breeding program for VWO resistance due to the low resistance level to *Verticillium dahliae* of their progeny, such as ‘Arbosana’ and ‘Arbequina’ reciprocal crosses. Furthermore, both cultivars have good agronomic characteristics and are suitable for superhigh-density plantation systems [30]. Both characteristics make it necessary, when using these parents, to evaluate a larger number of seedlings to discard and make strict selections with the best genotypes.

According to this study, ‘Frantoio’ in open pollination and the reciprocal crosses coming from ‘Arbosana’ and ‘Koroneiki’ and ‘Arbosana’ and ‘Frantoio’ are some of the best crosses to obtain new resistant cultivars. Some of these cultivars have been previously reported to generate a higher proportion of resistant offspring than other cultivars [10,11]. To completely discard the lack of maternal effect in the inheritance of VWO resistance, it would be interesting to evaluate a similar set of progenies under field conditions and increase the number of crosses evaluated.

## 4. Materials and Methods

### 4.1. Plant Material

Six olive cultivars were selected as genitors for directed crosses due to their positive agronomical traits and commercial importance [1]. For instance, ‘Arbequina’, ‘Arbosana’ and ‘Sikitita’ are widely used in superhigh-density olive orchards due to their productivity and low vigor, while ‘Koroneiki’ is highly appreciated because of its oil quality and relatively low vigor [37]. Moreover, these selected cultivars have been previously classified as resistant (‘Frantoio’), moderately susceptible (‘Arbequina’, ‘Arbosana’ and ‘Koroneiki’) and susceptible (‘Picual’) to infections caused by *V. dahliae* according to previous evaluations conducted under controlled [7,38,39] and field conditions [8].

Directed crosses of these olive cultivars were performed in the spring of 2016 and 2018 in trees of the World Olive Germplasm Bank of Cordoba-UCO Collection [40]. In 2016, we performed seven reciprocal crosses resulting from crossing in both directions ‘Arbosana’ × ‘Koroneiki’, ‘Arbosana’ × ‘Frantoio’, ‘Arbosana’ × ‘Picual’, ‘Arbosana’ × ‘Sikitita’, ‘Arbosana’ × ‘Arbequina’, ‘Picual’ × ‘Frantoio’ and ‘Sikitita’ × ‘Frantoio’ (Table 2). In addition, the offspring of ‘Picual’ and ‘Frantoio’ in open pollination were included because they represented the widest range of variability coming from a susceptible and a resistant cultivar. They have also been evaluated in previous studies, as well as the crossing ‘Frantoio’ × ‘Picual’ and its reciprocal [11]. In 2018, we conducted the crosses ‘Arbosana’ × ‘Sikitita’, ‘Picual’ × ‘Frantoio’ and their reciprocals, along with ‘Frantoio’ and ‘Picual’ in open pollination, with a higher number of genotypes to confirm the results obtained in 2016 (Table 2).

Directed crosses between cultivars and the germination of their offspring were performed according to Rallo et al. [31] by applying male pollen to female bagged branches [41]. Naked seeds from the resulting fruits, harvested in October 2016 and 2018, were stratified in cell trays filled with a mix of blond peat moss (40%), coconut fiber (30%), substratum (15%), and perlite (15%) at 13 to 14 °C and a relative humidity (RH) of 95% under dark conditions in a climatic chamber. A total of 1992 seeds were sown, sowing between 52 and 206 seeds per cross depending on the availability of seeds (Table 1).

After 30 days, we changed the parameters of the climatic chamber to 24 °C, 70% RH and continuous light for 5 weeks. The percentage of germinated plants of each cross was calculated by counting the plants with fully expanded cotyledons five weeks after sowing (Table 1). When genotypes had between 3 or 4 pairs of true leaves, they were ready to be inoculated.

To verify that the crosses were not contaminated with alien pollen, microsatellite (SSR)-based paternity tests were performed to confirm the genitors of each progeny. To do so, we extracted DNA from the leaves of 10 plants per cross, and their SSR profiles were amplified and compared with those of their putative genitors according to the protocol established by Diaz et al. [42].

### 4.2. Fungal Material and Plant Inoculation

The V117 isolate, a defoliating pathotype of *Verticillium dahliae* from cotton, was used as a fungal material to inoculate the seedlings. This isolate belongs to the mycology library of the Agroforestry Pathology Unit of the Department of Agronomy of the University of Córdoba [43]. The V117 isolate was collected from infected cotton in southern Andalusia (Spain), and its high virulence was verified in several artificial inoculations [9,39]

The original monoconidic cultures of *V. dahliae* were conserved in middle Plum Extract Agar (AEC) at 4 °C and in total darkness. To obtain isolate V117, a small portion of mycelium was taken from the tubes of AEC, planted in PDA medium and incubated at 24 °C for 1–2 weeks in darkness. The margins of the resulting colony were transferred back to PDA. This Petri dish culture was used to obtain the inoculum, remaining active through transfers in PDA during the execution of all experiments. To obtain the inoculum of the V117 isolate, it was sown in portions of PDA with mycelium on Petri plates and incubated for 7 days at 24 °C.

Plants with at least two pairs of true leaves were inoculated by dipping their bare root systems for 30 min in a conidial suspension of the pathogen adjusted to 10^7^ conidia/mL according to Trapero et al. [9]. All reciprocal crosses were inoculated together in the conidial suspension to homogenize the response. Controls were treated the same, but sterilized water was used instead of the inoculum.

### 4.3. Fungus Isolation

Plant infection was confirmed by isolating the fungus from the affected shoots of diseased plants. Affected woody tissue samples collected from infected seedlings were washed in running tap water. The tissue surface was then disinfected in 0.5% sodium hypochlorite for 45 s. Small pieces of nonbark stem were placed on PDA plates and incubated at 24 °C in the dark for 6 days.

### 4.4. Experimental Design

The experiments were independently carried out in 2016 with seven reciprocal crosses and two reciprocal crosses in 2018 applying the same methodology. Germinated plants were grown in a controlled environment chamber for 3 months after inoculation with continuous light at 24 °C and 60–80% RH. A completely randomized block design was applied in both years. In 2016, we included 8 blocks of 44 inoculated plants each (44 × 8 = 352 plants) and 4 blocks with 31 control plants (not inoculated) each (31 × 4 = 124) (Table 2). In total, we evaluated 352 inoculated plants and 124 control or noninoculated plants, including all crosses. In 2018, we included 5 blocks of 31 inoculated plants each and 4 blocks of 13 noninoculated control plants each, resulting in a total of 160 inoculated and 52 noninoculated plants.

### 4.5. Disease Evaluation

The symptoms were evaluated weekly for 13 weeks after inoculation. Disease severity was evaluated using a 0 to 100 rating scale. This scale estimated the percentage of affected aerial plant tissue in four main categories or quarters (<25, 26–50, 51–75, and 76–100%) with four values per category. Thus, each scale value represented the number of sixteenths of affected plant areas. The scale values (X) were linearly related to the percentage of affected tissue (Y) by the equation Y = 6.25X − 3.125 [44].

These values were used to build progress curves for the DI of the affected plants and the mean severity of the symptoms over time and to obtain the final mean severity value. The RAUDPC was estimated as the percentage of the maximum possible value in the considered period according to the formula based on Campbell and Madden [45]: AUDPC = [(t/2 × (S2 + 2 × S3 + … + 2Si − 1 + Si) / 4 × n] × 100, where t = the interval in days between observations; Si = the final mean severity; 4 = the maximum disease rating; and n = the number of observations. Mortality (M) or final percentage of dead plants was estimated with the higher value of severity.

### 4.6. Plant Growth Evaluation

Plant growth after inoculation was assessed in all genotypes by measuring the height of the plants at inoculation time and then every two weeks using a ruler. With these data, the average increase in height over time was estimated in noninoculated plants and inoculated plants without symptoms at the end of the experiment (GI). The GI was calculated by subtracting the final measurement from the height on the day of inoculation or the initial height.

### 4.7. Statistical Analysis

An association chi-square test using multiple comparisons for proportions with *p* = 0.05 was used to evaluate germination (%), DI (%) and M (%). Once the homogeneity values of variance and normality were verified, an analysis of variance (ANOVA) was performed with disease severity, RAUDPC and GI. The mean values of the analyzed parameters were compared using Fisher’s protected least significant differences test at *p* = 0.05. The program used in all statistical analyses was the Statistix 10.0 software program (Analytical Software, Tallahassee, FL, USA).

## Figures and Tables

**Table 1:** Seed number and average germination rate (%) per reciprocal cross in the 2016 and 2018 experiments.

Crosses	Seeds	Germination ^1^
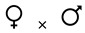	(n°)	(%)
‘Arbosana’ × ‘Koroneiki’	104	54.8 bcd
‘Koroneiki’ × ‘Arbosana’	104	56.7 bcd
‘Arbosana’ × ‘Frantoio’	104	68.3 cd
‘Frantoio’ × ‘Arbosana’	104	56.7 bcd
‘Picual’ × ‘Frantoio’	104	76.0 de
‘Frantoio’ × ‘Picual’	104	62.5 bcd
‘Arbosana’ × ‘Sikitita’	104	29.8 a
‘Sikitita’ × ‘Arbosana’	104	42.3 ab
‘Arbosana’ × ‘Arbequina’	104	29.8 a
‘Arbequina’ × ‘Arbosana’	104	50.0 abc
‘Frantoio’ open pollination	52	55.8 bcd
‘Picual’ open pollination	52	48.6 ab
Mean 2016	1144	52.6
‘Picual’ × ‘Frantoio’	160	56.3 b
‘Frantoio’ × ‘Picual’	152	36.2 ab
‘Arbosana’ × ‘Sikitita’	206	39.3 ab
‘Sikitita’ × ‘Arbosana’	156	23.7 a
‘Frantoio’ open pollination	81	72.8 bc
‘Picual’ open pollination	93	80.6 c
Mean 2018	848	51.5
Total	1992	52.2

^1^ Germination values followed by the same letter are not significantly different according to a chi-square test (*p* = 0.05).

**Table 2:** Crosses, seedling number, main disease values and growth in the 2016 and 2018 experiments comparing between reciprocal crosses.

Crosses ^1^	Seedlings	Disease Incidence ^2^	Final Severity ^3^	RAUDPC ^3^	Mortality ^2^	Growth ^3^ (cm)	
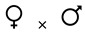	(n)	(%)	(%)	(%)	(%)	Inoculated	Control
‘Arbosana’ × ‘Koroneiki’	34	32.4	19.1 c	10.4 d	2.9	10.1 cd	20.6 a
‘Koroneiki’ × ‘Arbosana’	36	41.7	23.6 bc	14.4 cd	5.5	9.8 d	18.0 a
‘Arbosana’ × ‘Frantoio’	40	35.0	23.1 bc	11.2 d	12.5	11.4 bcd	18.4 a
‘Frantoio’ × ‘Arbosana’	36	47.2	28.5 abc	18.2 bcd	19.4	12.5 abcd	19.0 a
‘Picual’ × ‘Frantoio’	42	35.7	30.3 abc	21.8 abcd	26.2	12.4 abcd	16.1 a
‘Frantoio’ × ‘Picual’	32	21.9	18.7 c	11.8 d	12.5	13.9 abc	19.5 a
‘Arbosana’ × ‘Sikitita’	18	58.8	44.1 ab	30.1 abc	35.3	16.0 ab	18.0 a
‘Sikitita’ × ‘Arbosana’	26	50.0	39.4 abc	29.8 ab	34.6	15.8 ab	19.1 a
‘Arbosana’ x’ Arbequina’	16	62.5	49.3 a	38.1 a	43.8	15.3 ab	16.4 a
‘Arbequina’ × ‘Arbosana’	34	61.8	50.0 a	29.1 ab	35.3	12.0 abcd	16.6 a
‘Frantoio’ free pollination	16	25.0	15.62 c	12.3 d	6.3	16.2 a	19.3 a
‘Picual’ free pollination	22	45.5	37.5 abc	30.1 ab	22.7	14.0 abc	21.5 a
Mean 2016	352	43.1	31.7	21.4	21.4	13.28	18.5
‘Arbosana’ × ‘Sikitita’	36	66.7	57.5 a	33.3 a	44.4	13.4 a	16.9 a
‘Sikitita’ × ‘Arbosana’	16	56.3	52.5 ab	34.4 a	50	16.5 a	16.6 a
‘Picual’ × ‘Frantoio’	36	44.4	30.0 bc	13.5 b	16.7	12.3 a	19.0 a
‘Frantoio’ × ‘Picual’	20	57.9	50.0 ab	20.3 ab	31.6	16.3 a	19.8 a
‘Frantoio’ open pollination	20	21.1	17.5 c	6.9 b	5.3	14.4 a	16.4 a
‘Picual’ free pollination	32	41.9	30.0 bc	14.4 b	12.9	16.2 a	16.2 a
Mean 2018	160	48.0	39.6	20.5	26.8	14.85	17.5
Total	512	44.8	34.3	21.1	23.2	13.8	18.1

^1^ The experiment was repeated two times: in 2016 and 2018. In 2016, 12 crosses were evaluated, and in 2018, 6 crosses were evaluated. ^2^ Values from the pair of reciprocal crosses are not significantly different according to the chi-square test at *p* = 0.05. ^3^ Values followed by the same letter are not significantly different according to LSD testing at *p* = 0.05.

**Table 3:** Average germination and disease values for the progeny of each pair of reciprocal crosses inoculated with *V. dahliae*.

Crosses	Incidence ^1^	Final Severity ^2^	RAUDPC ^2^	Mortality ^1^	Germination ^1^
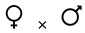	(%)	(%)	(%)	(%)	(%)
‘Frantoio’ open pollination	23.1 d	17.5 bcd	6.3 b	7.7 bc	55.8 abc
‘Arbosana’ × ‘Koroneiki’	37.1 bcd	22.5 cd	12.8 b	4.3 c	49.0 bc
‘Koroneiki’ × ‘Arbosana’
‘Arbosana’ × ‘Frantoio’	40.8 bcd	25.0 cd	14.5 b	15.8 bc	59.6 ab
‘Frantoio’ × ‘Arbosana’
‘Picual’ × ‘Frantoio’	29.8 cd	25.0 cd	17.5 b	20.3 ab	69.2 a
‘Frantoio’ × ‘Picual’
‘Sikitita’ × ‘Arbosana’	53.5 ab	42.5 ab	29.4 a	34.9 a	62.5 ab
‘Arbosana’ × ‘Sikitita’
‘Picual’ open pollination	43.4 abc	37.5 abc	32.3 a	21.7 ab	48.6 bc
‘Arbequina’ × Arbosana	62.0 a	50.0 a	33.1 a	38.0 a	36.1 c
‘Arbosana’ × ‘Arbequina’

^1^ Values from the pair of reciprocal crosses are not significantly different according to the chi-square test at *p* = 0.05. ^2^ Values followed by the same letter are not significantly different according to LSD testing at *p* = 0.05.

## Data Availability

Data available on request due to restrictions.
